# The positivity offset theory of anhedonia in schizophrenia: evidence for a deficit in daily life using digital phenotyping

**DOI:** 10.1017/S0033291722003774

**Published:** 2023-10

**Authors:** Lisa A. Bartolomeo, Ian M. Raugh, Gregory P. Strauss

**Affiliations:** Department of Psychology, University of Georgia, Athens, GA, USA

**Keywords:** Ecological momentary assessment, emotional experience, hedonic capacity, negative symptoms, psychosis

## Abstract

**Background:**

Negative symptoms of schizophrenia have recently been proposed to result from a decoupling of (intact) hedonic experience and (diminished) approach behavior. The current study challenged this view by exploring the hypothesis that negative symptoms are driven by a specific type of emotional experience abnormality, a reduction in the positivity offset (i.e. the tendency to experience greater levels of positive relative to negative emotion in low-arousal contexts), which limits the production of approach behaviors in neutral environments.

**Methods:**

Participants included outpatients with SZ (*n* = 44) and healthy controls (CN: *n* = 48) who completed one week of active (ecological momentary assessment surveys of emotional experience and symptoms) and passive (geolocation, accelerometry) digital phenotyping. Mathematical modeling approaches from Cacioppo's Evaluative Space Model were used to quantify the positivity offset in daily life. Negative symptoms were assessed via standard clinical ratings, as well as active (EMA surveys) and passive (geolocation, accelerometry) digital phenotyping measures.

**Results:**

Results indicated that the positivity offset was reduced in SZ and associated with more severe anhedonia and avolition measured via clinical interviews and active and passive digital phenotyping.

**Conclusions:**

These findings suggest that current conceptual models of negative symptoms, which assume hedonic normality, may need to be revised to account for reductions in the positivity offset and its connection to diminished motivated behavior. Findings identify key real-world contexts where negative symptoms could be targeted using psychosocial treatments.

## Introduction

Deficits in motivation and pleasure have been considered core features of schizophrenia (SZ) since its initial conceptualization (Bleuler, 1911; Diefendorf & Kraepelin, 1907; Kraepelin, 1921). In psychiatrically healthy individuals, these processes are reciprocally connected, with motivational deficits leading to less frequent pleasurable experiences and hedonic deficits leading to reduced motivation for seeking out these experiences (Bradley & Lang, 2007). In contrast, emotional experience and behavior are decoupled in SZ, such that intact hedonic capacity fails to translate into volitional responding (Heerey & Gold, 2007). To explain this discrepancy, several conceptual models posit that negative symptoms result from dysfunctional cortico-striatal circuitry (Barch & Dowd, 2010; Kring & Barch, 2014; Strauss, Waltz, & Gold, 2013). Implicit among these models is the assumption that hedonic capacity is intact and deficits in multiple aspects of reward processing (e.g. reinforcement learning, effort-cost computation, value representation) that impact decision-making prevent intact hedonic responses from motivating approach behaviors. Although these models have been vital for our understanding of negative symptoms, they have not led to significant treatment breakthroughs, suggesting that our current mechanistic understanding is incomplete. One limitation of current models may be that the assumption of hedonic normality in SZ is premature, leading to a failure to adequately consider the role of emotional experience abnormalities in negative symptoms.

Caccioppo's seminal Evaluative Space Model (Cacioppo, 1999; Cacioppo & Berntson, 1994) presents a novel approach to understanding how emotional responses fail to generate motivated behavior in SZ. The ESM proposes that separate positive and negative evaluation systems evolved to guide motivated behavior. Both systems are characterized by an activation function representing the relationship between affective input into the system (i.e. arousal) and the resulting output (i.e. emotional response). Positive and negative activation functions are differentially calibrated to translate emotional responses from the affective system into adaptive motivated behaviors in specific contexts. The positive system is calibrated to respond with greater amounts of positive relative to negative emotion at lower levels of affective input, a function known as the ‘positivity offset.’ As affective input into the system increases, the negative system is calibrated to respond with greater levels of negative than positive emotion, a function termed the ‘negativity bias.’ (Cacioppo, 1999; Cacioppo & Berntson, 1994; Larsen, McGraw, & Cacioppo, 2001; Larsen, Norris, McGraw, Hawkley, & Cacioppo, 2009; Norris, Gollan, Berntson, & Cacioppo, 2010). Healthy individuals typically experience a greater balance of positive than negative emotion in most situations, which tend to be neutral and characterized by minimal affective input. The positivity offset is adaptive because it promotes exploratory behavior and approach of novel stimuli in neutral environments that allows for the acquisition of new rewards and resources. The negativity bias is adaptive because it leads to withdrawal behavior at high levels of arousal that are characteristic of highly negative or dangerous environments.

Using mathematical approaches from the ESM, Strauss, Visser, Lee, and Gold (2017) used a laboratory-based paradigm to compare the positivity offset and negativity bias in adults with SZ and controls (CN). Participants made unipolar reports of positivity, negativity, and arousal in response to pleasant, unpleasant, and neutral images from the International Affective Picture System (IAPS) (Lang, Bradley, & Cuthbert, 1997). Following methods from Ito and Cacioppo (2005), two separate regression equations were used to quantify parameters used to calculate the positivity offset and negativity bias. The predictor in these equations represents the affective input into the evaluative system (i.e. self-reported arousal). The dependent variable is the resulting output from the affective system (i.e. self-reported levels of positivity or negativity). From these equations, the intercepts and slopes for the positivity and negativity functions were used to calculate the positivity offset and negativity bias, where a greater intercept for positivity relative to negativity reflects the prototypical positivity offset and a greater slope for negativity reflects the negativity bias. Results indicated that although individuals with SZ displayed hedonic normality, as reported in dozens of other studies (i.e. comparable positive emotion and arousal to pleasant stimuli between SZ and CN) (Cohen & Minor, 2010; Llerena, Strauss, & Cohen, 2012), emotional experience abnormalities were present in SZ and associated with clinically rated anhedonia. Specifically, individuals with SZ displayed a reduction in the positivity offset compared to CN, as indicated by lower intercept values for positive emotion and reductions in the positive/negative intercept difference score. Lower difference scores also predicted higher ratings of anhedonia. Importantly, the diminished positivity offset existed in the presence of intact *hedonic capacity*, as indicated by greater slope for positive emotion in SZ than CN (i.e. at highest levels of arousal when stimuli are most motivationally significant, SZ produce comparably greater positive emotional responses than CN). Riehle, Pillny, and Lincoln (2022) replicated these findings in an online community sample of individuals varying in trait psychotic-like experiences and anhedonia. Collectively, findings from these laboratory-based studies provide a novel explanation for deficits in motivated behavior among individuals with SZ-spectrum symptoms despite intact hedonic capacity. However, the link between the diminished positivity offset and reductions in motivated behavior has only been inferred and not yet demonstrated empirically. To fully test the hypothesis that reductions in approach behaviors are associated with reductions in the positivity offset (despite intact hedonic capacity), it is necessary to examine emotional experience and motivated behavior during daily life.

**Fig. 1.**
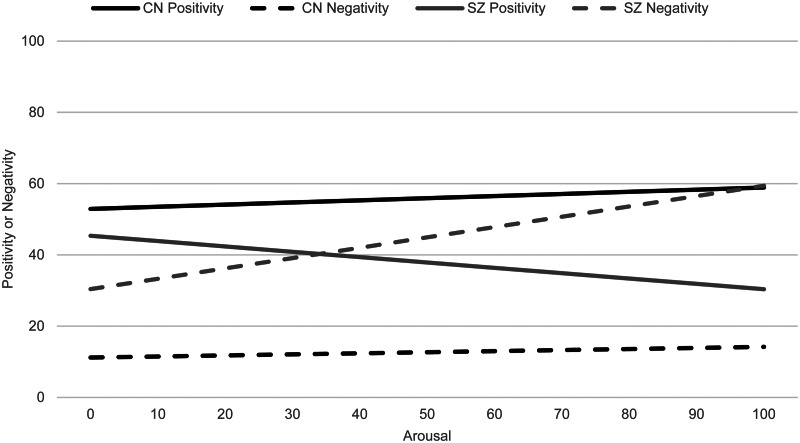
Positivity and negativity functions. *Note.* CN, control group; SZ, schizophrenia group. Affective input (i.e. arousal) is depicted on the *x*-axis and affective output (i.e. positivity or negativity) is depicted on the *y*-axis. The output when arousal = 0 represents the intercept of the positivity and negativity functions (i.e. the response of the affective system when input is absent). A greater intercept for positivity than negativity reflects the positivity offset, which activates approach motivation. The slopes of the lines represent the gain in positivity or negativity with increasing levels of arousal. A greater slope for negativity than positivity reflects the negativity bias, which activates withdrawal motivation.

The current study used active and passive digital phenotyping to determine whether the positivity offset is reduced in daily life and associated with greater self-reported and objectively quantified measures of anhedonia and avolition. Active digital phenotyping refers to measurements collected via mobile devices in the real-world that are purposefully triggered by the participant (e.g. surveys) (Onnela & Rauch, 2016), whereas passive digital phenotyping involves unobtrusively collecting data via sensors within a mobile device (e.g. geolocation, accelerometry) (Onnela & Rauch, 2016). Preliminary psychometric studies support the reliability and validity of active and passive digital phenotyping measures of negative symptoms in SZ, as well as their feasibility and tolerability (Depp et al., 2019; Fulford et al., 2021; Granholm et al., 2019; Harvey et al., 2021; Miller, Raugh, Strauss, & Harvey, 2022; Narkhede et al., 2021; Raugh et al., 2020; Raugh et al., 2021; Strauss et al., 2022). When used in tandem, active and passive digital phenotyping methods offer promise for exploring questions regarding the nature of emotion-motivation interactions in SZ since the same computational approaches validated for the ESM can be used in conjunction with objectively measured and self-reported behaviors.

The following hypotheses were made: (1) Based on prior laboratory-based findings (Strauss et al., 2017), participants with SZ will demonstrate a reduced positivity offset compared to CN on measures of active digital phenotyping (EMA surveys) collected in daily life; (2) Hedonic capacity measured via active digital phenotyping will be intact or elevated in SZ based on prior evidence for an increased slope for positivity relative to CN (Strauss et al., 2017); (3) Consistent with findings from Strauss et al. (2017), the negativity bias, measured via active digital phenotyping, will be intact in SZ; (4) Reductions in the active digital phenotyping-derived positivity offset difference score, but not the negativity bias, will be significantly associated with anhedonia and avolition measured via clinical rating scales, active digital phenotyping measures of negative symptoms in daily life, active digital phenotyping measures of the frequency of positive emotional experiences, and passive digital phenotyping measures of behavior obtained via geolocation and accelerometry.

## Method

### Participants

Forty-six individuals with DSM-5 (American Psychiatric Association, 2013) diagnoses of schizophrenia or schizoaffective disorder (SZ) and 50 psychiatrically healthy controls (CN) participated in the study. Two SZ and 2 CN participants were excluded for not reaching a priori digital phenotyping compliance standards (i.e. responding to < 20% of momentary surveys), resulting in a final sample of 44 SZ (16 with schizophrenia and 28 with schizoaffective disorder) and 48 CN. Groups did not significantly differ on age, sex, ethnicity, or parental education. SZ had lower personal education and momentary survey adherence rates than CN. Moderately severe symptoms and a typical magnitude of cognitive impairment were observed in SZ (see Table 1).

**Table 1.** Participant demographic and clinical characteristics

	SZ (*n* = 44)	CN (*n* = 48)	Test statistic	*p* value
Age	39.34 (12.02)	38.56 (10.50)	*F* *=* 0.11	0.74
Parental education	13.98 (2.83)	13.51 (2.86)	*F* *=* 0.58	0.40
Personal education	13.02 (2.27)	15.73 (2.82)	*F* *=* 25.46	< 0.001
Female (%)	63.60	72.90	χ^2^ *=* 0.92	0.34
Race (%)			χ^2^ *=* 7.19	0.21
Black	29.50	22.90		
Asian	0.00	6.30		
Latinx	4.50	12.50		
White	59.10	47.90		
Multiracial	6.80	6.30		
Other	0.00	4.20		
MCCB	41.91 (15.74)	52.40 (10.19)	*F* *=* 13.90	< 0.001
BNSS total	16.23 (13.66)	–		
BNSS avolition	2.10 (1.68)	–		
BNSS anhedonia	1.51 (1.51)	–		
BNSS asociality	1.50 (1.31)	–		
BNSS alogia	0.30 (0.91)	–		
BNSS blunted affect	1.16 (1.52)	–		
PANSS positive	2.57 (0.85)	–		
PANSS negative	1.89 (0.84)	–		
PANSS general	2.11 (0.51)	–		
Survey adherence rate	0.60 (0.23)	0.71 (0.20)	*F* *=* 5.84	0.02

*Note.* SZ, schizophrenia group; CN, control group. MCCB, MATRICS Consensus Cognitive Battery; BNSS, Brief Negative Symptom Scale; PANSS, Positive and Negative Syndrome Scale. BNSS and PANSS domain scores reflect average of item scores within each domain.

Individuals with SZ were recruited from local community outpatient mental health centers and advertisements. Clinical diagnoses were determined via the SCID-5 (First, 2015). CN were recruited from the local community using advertisements. CN were free of current major psychiatric diagnoses as established via the SCID-5, current SZ-spectrum personality disorders as established via the SCID-PD (First, Williams, Benjamin, & Spitzer, 2015), family history of psychosis, and psychotropic medications. All participants denied lifetime neurological disease and did not meet criteria for a substance abuse disorder within the last 6 months (excluding nicotine use disorders). Participants received monetary compensation for their participation and provided written informed consent for a protocol approved by the University of Georgia Institutional Review Board. Participants were compensated $20 per hour for laboratory sessions, $1 per mobile survey completed, and $80 for returning the phone at the end of the study.

### Procedure

The study consisted of three phases: (1) initial laboratory visit; (2) six consecutive days of digital phenotyping; and (3) final laboratory visit.

#### Phase 1: Initial laboratory visit

Clinical interviews were conducted to assess diagnoses and symptoms. Diagnostic and symptom interviews for SZ consisted of the SCID-5 (First, 2015) and Brief Negative Symptom Scale (BNSS; Kirkpatrick et al., 2011). CN interviews included the SCID-5 (First, 2015), and SCID-PD (First et al., 2015). All interviews were conducted by either Dr Strauss or lab personnel trained to reliability standards (inter-rater reliability of alpha > 0.80) who established consensus for diagnoses.

Participants were trained on digital phenotyping procedures and provided with a Blu Vivo 5R smartphone running Android operating system 7.0 programmed with the mEMA app from ilumivu to collect digital phenotyping data. Trained lab personnel instructed participants in the use of the phone and the mobile app, including a guided demonstration of survey notifications and a practice survey that provided an overview and explanation of the types of questions that would be asked. Participants were also trained on how to use and charge the Empatica wristband.

#### Phase 2: Digital phenotyping

*Active Digital Phenotyping***.** Over the 6-day digital phenotyping phase, participants were prompted with eight momentary surveys per day that were quasi-randomly scheduled within 90-minute epochs between 9 AM and 9 PM. Surveys were scheduled between 18 min to 3 h apart from each other. Attempts to respond to the survey after a 15-min window were not permitted, but participants were allotted unlimited time to complete the questions. Surveys assessed the following:

*Momentary Emotional Experience**.*** Every survey probed current levels of positive and negative emotion using the modified Differential Emotions Scale (mDES; Fredrickson, Tugade, Waugh, and Larkin, 2003). Each prompt assessed five negative (anger, fear, sadness, shame, anxiety) and five positive emotions (amused, content, happy, love, pride) rated on a 0–100 sliding scale anchored between ‘Not at all’ and ‘Extremely.’ Participants also identified whether their current emotional context was positive, negative, neutral, or mixed. Context responses were used to determine the frequency of positive experiences endorsed over the digital phenotyping period.

*Momentary Emotional Arousal.* Every survey probed current emotional arousal by asking ‘How keyed-up or excited are you right now?’. Participants rated arousal on a 0–100 sliding scale anchored between ‘Not at all’ and ‘Extremely.’

*Negative Symptoms.* Momentary surveys probed for negative symptoms of anhedonia, avolition, and asociality. Anhedonia was measured by averaging across momentary responses for consummatory (i.e. ‘How much are you enjoying the activity?’ and ‘How much are you enjoying this social interaction?’) and anticipatory pleasure (i.e. ‘How much do you think you will enjoy that activity the next time you do it?’ and ‘How much do you think you will enjoy interacting with them next time?’). Avolition was measured by assessing participants' level of interest in a current activity (i.e. ‘How interested are you in the activity?’). If participants reported they were not engaged in an activity (i.e. doing ‘Nothing.’), avolition was measured via desire to engage in an activity (i.e. ‘How much do you want to be doing an activity right now?’). Lastly, asociality was measured by assessing participants' interest in a social interaction (i.e. ‘How interested are you in this social interaction?’). If participants denied interacting with anyone, asociality was measured via responses about their desire to interact with others (i.e. ‘How much do you want to be interacting with someone right now?’). All items were rated on a 0 (not at all) to 100 (extremely) scale. These items have shown convergent validity via associations with clinical ratings of the same domains on the BNSS; confirmatory factor analysis also indicates that the items constitute three separate factors: anhedonia, avolition, asociality (Raugh et al., 2020).

*Infrequent Responding.* To monitor infrequent responding, a question from the Chapman Anhedonia Scale (Eckblad, Chapman, Chapman, & Mishlove, 1982) was embedded within each momentary survey. Participants responded ‘True’ or ‘False’ to the items, which portrayed common, every day experiences (e.g. ‘Sometimes when walking down the sidewalk, I have seen children playing.’; ‘I cannot remember a time when I talked with someone who wore glasses.’). The rate of infrequent responding was low (< 7%) in both groups.

*Passive Digital Phenotyping.* Geolocation was passively measured throughout the digital phenotyping phase via the smartphone. Geolocation involves collecting GPS coordinates at predetermined intervals or every time the participant moves a certain radius in a space. Phone sensors were programmed to collect geolocation every 10 min or when participants moved more than 10 m. To index change in geolocation, distance from home and percentage of time at home were extracted as the primary variables of interest given face validity as measures of goal-directed exploratory behavior and the growing use of these variables in prior studies. Secondary geolocation variables were calculated for exploratory purposes (i.e. number of locations, location variance, time spent in different locations, number of flights, transition time) that were previously validated in relation to negative symptoms (Granholm et al., 2019; Raugh et al., 2020).

Using the mEMA application, sensors within the study phones were programmed to measure accelerometry with each change in XYZ coordinate motion (every change in accelerometry being logged as a single instance), with separate values output for *X*, *Y*, and *Z* movement axes. Participants wore an Empatica wristband that collected accelerometry as a gravitational force (g units) at 32 Hz between −16 g and 16 g. Accelerometry has shown convergent validity with clinically rated negative symptoms (Strauss et al., 2022). See Table 2 for information on geolocation and acceleration variables and Table 3 for descriptive statistics and group comparisons.

**Table 2.** Geolocation and accelerometry variable definitions

GPS or ACL	Variable	Abbreviation	Definition
GPS	Home time	Home	Amount of time at home
GPS	Distance change	Δd MPD Range	Distance traveled in meters from previous sample Total meters traveled per day Range of distance per day
GPS	Distance from home	Δmh maxmh	Meters from home for each sample Maximum distance from home per day
GPS	Stationary location clusters	nc	Number of meaningful locations
GPS	Location variance	lv	Variance within a specified timeframe: lv = log[(*σ*2 lat + 1) + (*σ*2 long + 1)]
GPS	Entropy and normalized entropy	ent nent	Equity of time spent in different locations
GPS	Transition time	tt	Percentage of samples taken in transit
GPS	Flights	f.dur f.dist f.num	Average duration of discrete trips per day Average distance of discrete trips per day Average number of discrete trips per day
ACL	ACL mean	ACLB.mean	Accelerometry band mean
ACL	ACL variance	ACLB.s.d.	Accelerometry band average standard deviation

*Note.* GPS, geolocation; ACL, accelerometry.

**Table 3.** One-way ANOVAs comparing passive digital phenotyping variables between groups

Variable	Abbreviation	SZ	CN	Test statistic
Home time	Home	0.60 (0.29)	0.45 (0.22)	*F*_(__1, 76)_ = 5.97, *p* = 0.02, 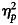 = 0.07
Distance change	Δd MPD Range	67.74 (76.44) 9033.09 (9543.91) 1202.92 (11 822.85)	68.93 (92.87) 9092.60 (9486.33) 28 934.47 (64 540.62)	*F*_(__1, 77)_ = 0.004, *p* = 0.95, 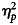 = 0 *F*_(1, 71)_ = 0.001, *p* = 0.98, 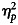 = 0 *F*_(1, 75)_ = 2.40, *p* = 0.13, 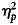 = 0.03
Distance from home	Δmh maxmh	6177.27 (11 581.12) 13 876.34 (19 382.35)	12 785.67 (15 852.94) 20 339.53 (21 115.99)	*F*_(__1, 76)_ = 4.30, *p* = 0.04, 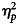 = 0.05 *F*_(1, 74)_ = 1.89, *p* = 0.17, 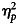 = 0.03
Stationary location clusters	nc	3.36 (1.15)	4.33 (1.95)	*F*_(__1, 74)_ = 6.76, *p* = 0.01, 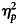 = 0.09
Location variance	lv	0.70 (0.01)	0.72 (0.15)	*F*_(__1, 73)_ = 1.12, *p* = 0.30, 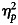 = 0.02
Entropy and normalized entropy	ent nent	0.21 (0.12) 0.18 (0.07)	0.23 (0.15) 0.16 (0.07)	*F*_(__1, 74)_ = 0.51, *p* = 0.48, 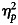 = 0.01 *F*_(1, 74)_ = 1.34, *p* = 0.25, 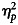 = 0.02
Transition time	tt	0.18 (0.14)	0.21 (0.12)	*F*_(__1, 75)_ = 1.04, *p* = 0.31, 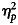 = 0.01
Flights	f.dur f.dist f.num	10.23 (14.85) 3267.51 (4176.62) 0.91 (0.79)	14.40 (16.66) 3991.39 (5636.92) 1.23 (0.65)	*F*_(__1, 75)_ = 1.31, *p* = 0.26, 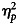 = 0.02 *F*_(1, 75)_ = 0.40, *p* = 0.53, 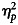 = 0.01 *F*_(1, 75)_ = 3.97, *p* = 0.05, 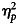 = 0.05
ACL mean	ACLB.mean	0.91 (0.06)	0.93 (0.11)	*F*_(__1, 52)_ = 0.45, *p* = 0.50, 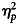 = 0.01
ACL variance	ACLB.s.d.	0.14 (0.02)	0.22 (0.49)	*F*_(__1, 52)_ = 0.62, *p* = 0.44, 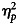 = 0.01

*Note.* SZ = schizophrenia group; CN = control group. Values reflect mean (s.d.) unless otherwise indicated. Refer to Table 2 for variable definitions.

#### Phase 3: Final laboratory visit

The final laboratory visit occurred one week after the initial laboratory visit, at the end of the digital phenotyping protocol. Participants returned the phone to the lab, completed the MATRICS Consensus Cognitive Battery (MCCB; Nuechterlein et al., 2008), and other procedures not reported in the current study.

### Data analysis

All analyses were conducted using SPSS v.27 except for multi-level supplemental analyses performed in R. Methods for calculating the positivity offset and negativity bias are based on Ito and Cacioppo (2005). The positivity offset and negativity bias are characterized by regression parameters where the positivity offset is represented as the intercept for positivity (the output at zero input) and the negativity bias represented as the slope for negativity (greater rate of change in output per unit of input). Positivity offset and negativity bias were assessed across participants within each group. Two regression analyses were conducted for each subject using the equation *E* = *Ax* + *b*, where *E* is either unipolar positivity or negativity ratings and *A* is the mean arousal rating. To model the positive motivational system, the intercept value derived from the equation represents the strength of the positivity offset, where positivity offset scores (i.e. the positive – negative intercept difference score) were calculated from multiple regression conducted on each participant and used to obtain the intercept score for positive and negative emotion for each day. To model the negative motivational system, the slope derived from the equation represents the strength of the negativity bias, which reflects the magnitude of increase in negative emotion output per unit of increase in affective input. The negativity bias difference score was calculated as the difference between the negativity and positivity slopes (i.e. negativity slope – positivity slope). The average intercept and slope difference scores were then calculated across all days and summarized at the level of the week to obtain the most reliable estimate. These difference scores then served as the dependent variables for primary analyses used to examine group differences and correlations. See Table 4 for a summary of the ESM variables.

**Table 4.** Evaluative space model definitions and formulas

Variable	Definition	Equation
Positivity intercept	Positive affective output when affective input is absent	*b* in the equation *E* *=* *Ax* *+* *b* where *E* *=* unipolar positivity rating and *A* = mean arousal rating
Positivity slope	Change in positive affective output per 1 unit change in arousal	*x* in the equation *E* *=* *Ax* *+* *b* where *E* *=* unipolar positivity rating and *A* = mean arousal rating
Negativity intercept	Negative affective output when affective input is absent	*b* in the equation *E* *=* *Ax* *+* *b* where *E* *=* unipolar negativity rating and *A* = mean arousal rating
Negativity slope	Change in negative affective output per 1 unit change in arousal	*x* in the equation *E* *=* *Ax* *+* *b* where *E* *=* unipolar negativity rating and *A* = mean arousal rating
Positivity offset	Greater positive affective output than negative affective output when affective input is absent leading to approach motivation	Positivity intercept – Negativity intercept
Negativity bias	Greater gain in negative affective output than positive affective output with increasing levels of affective input leading to withdrawal motivation	Negativity slope – Positivity slope

*Note.* Adapted from Cacioppo, Berntson, Norris, & Gollan (2012).

Preliminary analyses of standard comparisons of valence and arousal, including effects of emotional context, were conducted as described in Strauss et al. (2017) and displayed in online Supplemental Materials. For primary analyses, within-group paired sample *t* tests were conducted comparing positive and negative intercepts and slopes to determine if each group demonstrated the prototypical positivity offset and negativity bias. Separate one-way ANOVAs were conducted on the 1-week average positivity offset intercept difference score, the negativity bias difference score, and the raw positivity and negativity intercept and slopes to assess group differences in positivity and negativity parameters. Raw intercept comparisons reflect group differences in how the positivity and negativity systems are calibrated to respond when affective input is absent, and the slope comparisons group differences in hedonic capacity. Pearson correlations were used to examine the relationship between positivity and negativity parameters with accelerometry and geolocation as measures of avolition in the SZ group (see Table 2 for digital phenotyping variables included in correlational analyses), as well as avolition and anhedonia measured via the BNSS and digital phenotyping. Additionally, the association between the number of positive experiences participants endorsed over the digital phenotyping period and the positivity offset difference score was examined using bivariate correlations.

Additional exploratory analyses examining the effects of sex, diagnosis (i.e. schizoaffective disorder *v.* schizophrenia), and associations with medication status, cognition, positive symptoms, and depressive symptoms are reported in online Supplemental Materials.

## Results

### Group comparisons of positivity and negativity parameters

Both SZ (*t* = 2.13, *p* = 0.04) and CN (*t* = 9.61, *p* < 0.001) demonstrated the positivity offset, with significantly higher intercepts for positivity than negativity. Slopes for the positivity function did not significantly differ between groups. As hypothesized, the positivity offset intercept difference score was significantly reduced in SZ compared to CN (*F*_(1, 92)_ = 10.86, *p* = 0.001, 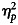 = 0.11). Neither group demonstrated the negativity bias, evidenced by nonsignificant differences between the slope for positivity and negativity in SZ (*t* = −1.69, *p* = 0.10) and CN (*t* = 0.52, *p* = 0.61). Groups did not significantly differ on the negativity bias slope difference score (see Table 5 and Figure 1).

### Correlations between positivity and negativity parameters and negative symptoms

In SZ, greater reductions in positivity offset were associated with reduced vigor of movement (i.e. ACLB.mean) (*r* = 0.53, *p* = 0.02) and greater variability in movement (i.e. ACLB.s.d.) (*r* = −0.52, *p* = 0.02) measured by the wristband (see Table 2 for digital phenotyping variable definitions). Greater variability in movement is more common among sedentary compared to active individuals, with the latter demonstrating steady fluctuations in movement and speed throughout the day. Past research suggests that individuals with SZ spend more time in sedentary than active contexts compared to CN (Strassnig et al., 2021), suggesting that this finding reflects the relationship between deficient activity and approach motivation in daily life. Lower positivity offset scores were associated with more severe avolition and anhedonia measured via the BNSS (avolition: *r* = −0.34, *p* = 0.03; anhedonia: *r* = −0.43, *p* < 0.01) and active digital phenotyping (avolition: *r* = −0.57, *p* < 0.001; anhedonia: *r* = −0.58, *p* < 0.001) in SZ. Lastly, greater reductions in the positivity offset were associated with a lower frequency of positive events in SZ (*r* = 0.34, *p* = 0.03). Correlations between the negativity bias score and clinically rated and active and passive digital phenotyping measures of avolition and anhedonia were all nonsignificant (*p*'s all > 0.05).

**Table 5.** One-way ANOVAs comparing positivity and negativity parameters in schizophrenia and control groups

	SZ	CN	Test statistic
Positivity intercept	45.38 (24.86)	52.91 (21.06)	*F*_(__1, 92)_ = 2.47, *p* = 0.12, 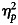 = 0.03
Negativity intercept	30.41 (30.25)	11.19 (12.60)	*F*_(__1, 92)_ = 16.30, *p* < 0.001, 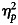 = 0.15
Positivity slope	−0.15 (1.06)	0.06 (0.26)	*F*_(__1, 92)_ = 1.72, *p* = 0.19, 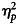 = 0.02
Negativity slope	0.29 (1.38)	0.03 (0.19)	*F*_(__1, 92)_ = 1.73, *p* = 0.19, 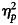 = 0.02
Positivity offset Difference score	14.97 (46.66)	41.72 (30.07)	*F*_(__1, 92)_ = 10.86, *p* = 0.001, 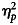 = 0.11
Negativity bias difference score	0.44 (1.73)	−0.03 (0.40)	*F*_(__1, 92)_ = 3.37, *p* = 0.07, 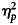 = 0.04

*Note.* SZ, schizophrenia group; CN, control group. Positivity Offset Difference Score = Positivity Intercept – Negativity Intercept. Negativity Bias Difference Score = Negativity slope –positivity slope. Values reflect mean (s.d.) unless otherwise indicated.

In SZ, lower raw positivity intercepts were significantly associated with more severe anhedonia (*r* *=* −0.36, *p* *=* *0*.02) and avolition (*r* *=* −0.32, *p* *=* *0*.03) measured via the BNSS and active digital phenotyping (anhedonia: *r* *=* −0.63, *p* *<* *0*.001; avolition: *r* *=* −0.57, *p* *<* *0*.001). Greater reductions in the positivity slope in SZ were significantly correlated with more severe clinically-rated anhedonia (*r* *=* −0.36, *p* *=* *0*.02). In SZ, higher intercepts for the negativity function were associated with more severe anhedonia (*r* *=* *0*.34, *p* *=* *0*.02) and avolition (*r* *=* *0*.41, *p* *=* *0*.01) measured via active digital phenotyping, as well as greater variability in movement measured by the wristband (i.e. ACLB.s.d.) (*r* *=* *0*.54, *p* *=* *0*.01). Correlations with secondary passive digital phenotyping variables used for exploratory purposes were all nonsignificant.

## Discussion

The current study aimed to determine if the positivity offset is reduced in the daily lives of people with SZ and associated with negative symptoms measured via clinical interviews and digital phenotyping. Consistent with several past laboratory-based and experience sampling studies, digital phenotyping results indicated that hedonic capacity is intact in SZ across positive, negative, and neutral contexts (Cohen & Minor, 2010; Gard & Kring, 2009; Gard, Kring, Gard, Horan, & Green, 2007; Gold, Waltz, Prentice, Morris, & Heerey, 2008; Kring & Moran, 2008). Conversely, emotional experience abnormalities emerged when the ESM was applied. Specifically, the positivity offset measured via active digital phenotyping was significantly reduced in SZ compared to CN, suggesting that patients experience reduced levels of positive relative to negative emotion at low levels of arousal. The nonsignificant group differences in the slope for the positivity function indicates that patients' positive emotions increase as arousal increases, suggesting that hedonic capacity is intact in SZ and hedonic deficits are only present when affective input is low. This extends laboratory-based evidence for the positivity offset reduction in SZ (Strauss et al., 2017) by demonstrating that it also occurs in the context of daily life, outside of a controlled laboratory setting with controlled emotional stimuli. Also extending past findings (Strauss et al., 2017), greater reductions in the positivity offset were associated with increased avolition and anhedonia measured via the BNSS and active and passive digital phenotyping. The reduced positivity offset was also associated with the frequency of positive experiences measured via the active digital phenotyping emotion context item (i.e. the behavioral component of anhedonia). Together, these findings extend past laboratory-based studies (Riehle et al., 2022; Strauss et al., 2017), providing a direct link between the positivity offset reduction and real-world behavior. The association between the positivity offset reduction and digital phenotyping measures of negative symptoms indicate that it may be a relevant target for improving deficits in approach behavior in SZ. Further, results indicated that the negativity bias is intact in SZ during everyday activities and unrelated to any measures of avolition or anhedonia. These findings were also consistent with prior laboratory evidence (Strauss et al., 2017), suggesting that within the ESM, negative symptoms were associated with the positivity offset rather than the negativity bias.

The current findings should be considered in the context of certain limitations. First, the positivity offset and negativity bias were calculated based on subjective reports of arousal and emotion. Future laboratory-based and digital phenotyping studies should incorporate physiological measures of arousal and emotional responding (e.g. heart rate variability, skin conductance, pupil dilation) to further understand abnormalities within the context of the ESM, including those at the neural level. Research in psychiatrically healthy individuals indicates that genetic polymorphisms involved in the serotonin system and other neuromodulatory genes may influence the magnitude of the positivity offset (Ashare, Norris, Wileyto, Cacioppo, & Strasser, 2013; Norris, Larsen, Crawford, & Cacioppo, 2011). fMRI studies are also necessary for identifying brain regions implicated in the diminished positivity offset in SZ, such as abnormalities within the limbic system and reward circuitry (Costa, Lang, Sabatinelli, Versace, & Bradley, 2010; Norris et al., 2011). Second, the accelerometry and geolocation variables included in the study belong to the ‘third generation’ of negative symptom assessments that are still being validated. Additional work is needed to extend findings from preliminary validation studies (Narkhede et al., 2021; Raugh et al., 2020; Strauss et al., 2022) and identify which are the strongest, most reliable measures of negativity symptoms. Third, the current sample included adult outpatients with chronic, stable SZ. Thus, it is unclear if the results would extend to earlier stages of illness and those with greater symptom severity. Finally, a clinical comparison group was not included. Past laboratory-based behavioral findings indicate that the positivity offset is reduced in individuals with depression (Gollan et al., 2016), who also commonly experience avolition and anhedonia. This may suggest that the positivity offset is not specific to SZ and could be a transdiagnostic mechanism underlying avolition and anhedonia. Further, given that the association between the positivity offset and depression was also significant (see online Supplemental Materials), future studies should examine the role of both primary and secondary negative symptom factors.

Findings have important clinical implications. Interventions like behavioral activation and activity scheduling may effectively increase the positivity offset and improve avolition or anhedonia. These approaches are main components of Negative Symptom Focused Cognitive Behavior Therapy (Perivoliotis, Grant, & Beck, 2010), and past studies indicated that they are feasible and effective at reducing negative symptoms in SZ (Choi, Jaekal, & Lee, 2016; Grant, Huh, Perivoliotis, Stolar, & Beck, 2012; Lee et al., 2018; Mairs, Lovell, Campbell, & Keeley, 2011); however, no study to date has examined the relationship between behavioral activation, the positivity offset, and deficits in motivation and pleasure in SZ. Mobile health (mHealth) interventions may provide an alternative way to target real-world impairments in hedonic and motivational processes from the perspective of the ESM. For example, mHealth apps could be programmed to assist in activity scheduling, including sending reminders for activities, and to notify patients to become behaviorally activated in neutral contexts in a way that will provide opportunities to enhance positive emotion. Passive digital phenotyping could be directly incorporated into treatment, such as sending notifications to become behaviorally activated when objective behavioral markers (e.g. speed of movement, activity index) fall below a relevant threshold. Emotion regulation interventions delivered via in-person therapy and/or mHealth may also help patients to more effectively increase positive emotion and decrease negative emotion as a means of normalizing the positivity offset to facilitate motivated behavior. Emotion regulation strategies like savoring and reappraisal may be particularly beneficial for increasing positive emotion and decreasing negative emotion (Favrod et al., 2019).

In conclusion, the present findings support the hypothesis that the diminished positivity offset is associated with negative symptoms in SZ. These results refute prior assumptions of hedonic normality and affect-behavior decoupling. Instead, deficits in motivated behavior appear to be driven by an imbalance in positive relative to negative affect in low arousal contexts. The specificity of when affective abnormalities drive motivated behavior deficits, at low levels of arousal, should be used to personalize novel treatment approaches to everyday contexts where avolition and anhedonia are most relevant. Pending replication and extension, conceptual models of negative symptoms should incorporate affective abnormalities like the positivity offset reduction, in addition to dysfunctional reward processing, as an important process leading to negative symptoms.

## Supplementary material

For supplementary material accompanying this paper visit https://doi.org/10.1017/S0033291722003774. click here to view supplementary material

## References

[ref1] American Psychiatric Association, D.-T. F. (2013). Diagnostic and statistical manual of mental disorders: DSM-5™ (5th ed.). Arlington, VA, USA: American Psychiatric Publishing, Inc.

[ref2] Ashare, R. L., Norris, C. J., Wileyto, E. P., Cacioppo, J. T., & Strasser, A. A. (2013). Individual differences in positivity offset and negativity bias: Gender-specific associations with two serotonin receptor genes. Personality and Individual Differences, 55(5), 469–473. doi: 10.1016/j.paid.2013.04.009 23976810 PMC3747009

[ref3] Barch, D. M., & Dowd, E. C. (2010). Goal representations and motivational drive in schizophrenia: The role of prefrontal-striatal interactions. Schizophrenia Bulletin, 36(5), 919–934. doi: 10.1093/schbul/sbq068 20566491 PMC2930335

[ref4] Bleuler, E. (1911). Dementia Praecox or the group of schizophrenias. New York: International Universities Press Inc.

[ref5] Bradley, M. M., & Lang, P. J. (2007). Emotion and motivation. In J. T. Cacioppo, L. G. Tassinary, & G. G. Berntson (Eds.), Handbook of psychophysiology (3rd ed., pp. 581–607). New York, NY, USA: Cambridge University Press.

[ref6] Cacioppo, J. T. (1999). The affect system Has parallel and integrative processing components form follows function. Journal of Personality and Social Psychology, 76(5), 839–855. Retrieved from https://psycnet.apa.org/record/1999-13561-011.

[ref7] Cacioppo, J. T., & Berntson, G. G. (1994). Relationship between attitudes and evaluative space: A critical review, with emphasis on the separability of positive and negative substrates. Psychological Bulletin, 115(3), 401–423. doi: 10.1037/0033-2909.115.3.401

[ref8] Choi, K.-H., Jaekal, E., & Lee, G.-Y. (2016). Motivational and behavioral activation as an adjunct to psychiatric rehabilitation for mild to moderate negative symptoms in individuals with schizophrenia: A proof-of-concept pilot study. Frontiers in Psychology, 7, 1759. doi: 10.3389/fpsyg.2016.01759 PMC5107574 27895602

[ref9] Cohen, A. S., & Minor, K. S. (2010). Emotional experience in patients with schizophrenia revisited: Meta-analysis of laboratory studies. Schizophrenia Bulletin, 36(1), 143–150. doi: 10.1093/schbul/sbn061 18562345 PMC2800132

[ref10] Depp, C. A., Bashem, J., Moore, R. C., Holden, J. L., Mikhael, T., Swendsen, J., … Granholm, E. L. (2019). GPS Mobility as a digital biomarker of negative symptoms in schizophrenia: A case control study. npj Digital Medicine, 2(1), 108. doi: 10.1038/s41746-019-0182-1 31728415 PMC6841669

[ref11] Diefendorf, A. R., & Kraepelin, E. (1907). Clinical psychiatry: A textbook for students and physicians, abstracted and adapted from the 7th German edition of Kraepelin's Lehrbuch der Psychiatrie. New York, NY, USA: MacMillan Co.

[ref12] Favrod, J., Nguyen, A., Chaix, J., Pellet, J., Frobert, L., Fankhauser, C., … Bonsack, C. (2019). Improving pleasure and motivation in schizophrenia: A randomized controlled clinical trial. Psychotherapy and Psychosomatics, 88(2), 84–95. doi: 10.1159/000496479 30783071 PMC6518864

[ref13] First, M. B. (2015). Structured Clinical Interview for the DSM (SCID). *In R. L. Cautin & S.O. Lilienfeld (Eds.)*, The encyclopedia of clinical psychology.

[ref14] First, M. B., Williams, J. B. W., Benjamin, L. S., & Spitzer, R. L. (2015). User's guide for the SCID-5-PD *(*Structured Clinical Interview for DSM-5 Personality Disorder*)*. Arlington, VA: American Psychiatric Association.

[ref15] Fredrickson, B. L., Tugade, M. M., Waugh, C. E., & Larkin, G. R. (2003). What good are positive emotions in crises? A prospective study of resilience and emotions following the terrorist attacks on the United States on September 11th, 2001. Journal of Personality and Social Psychology, 84(2), 365–376. doi: 10.1037//0022-3514.84.2.365 12585810 PMC2755263

[ref16] Fulford, D., Mote, J., Gonzalez, R., Abplanalp, S., Zhang, Y., Luckenbaugh, J., Onnela, … Gard, D. E. (2021). Smartphone sensing of social interactions in people with and without schizophrenia. Journal of Psychiatric Research, 137, 613–620. doi: 10.1016/j.jpsychires.2020.11.002 33190842 PMC8084875

[ref17] Gard, D. E., & Kring, A. M. (2009). Emotion in the daily lives of schizophrenia patients: Context matters. Schizophrenia Research, 115(2–3), 379–380. doi: 10.1016/j.schres.2009.07.017 19695838

[ref18] Gard, D. E., Kring, A. M., Gard, M. G., Horan, W. P., & Green, M. F. (2007). Anhedonia in schizophrenia: Distinctions between anticipatory and consummatory pleasure. Schizophrenia Research, 93, 253–260. doi: 10.1016/j.schres.2007.03.008 17490858 PMC1986826

[ref19] Gold, J. M., Waltz, J. A., Prentice, K. J., Morris, S. E., & Heerey, E. A. (2008). Reward processing in schizophrenia: A deficit in the representation of value. Schizophrenia Bulletin, 34(5), 835–847. doi: 10.1093/schbul/sbn068 18591195 PMC2518641

[ref20] Gollan, J. K., Hoxha, D., Hunnicutt-Ferguson, K., Norris, C. J., Rosebrock, L., Sankin, L., & Cacioppo, J. (2016). Twice the negativity bias and half the positivity offset: Evaluative responses to emotional information in depression. Journal of Behavior Therapy and Experimental Psychiatry, 52, 166–170. doi: 10.1016/j.jbtep.2015.09.005 26434794 PMC5685183

[ref21] Granholm, E., Holden, J. L., Mikhael, T., Link, P. C., Swendsen, J., Depp, … Harvey, P. D. (2019). What do people with schizophrenia do all day? Ecological momentary assessment of real-world functioning in schizophrenia. Schizophrenia Bulletin, 46(2), 242–251. doi: 10.1093/schbul/sbz070 PMC7442321 31504955

[ref22] Grant, P. M., Huh, G. A., Perivoliotis, D., Stolar, N. M., & Beck, A. T. (2012). Randomized trial to evaluate the efficacy of cognitive therapy for low-functioning patients with schizophrenia. Archives of General Psychiatry, 69(2), 121–127. doi: 10.1001/archgenpsychiatry.2011.129 21969420

[ref23] Harvey, P. D., Miller, M. L., Moore, R. C., Depp, C. A., Parrish, E. M., & Pinkham, A. E. (2021). Capturing clinical symptoms with ecological momentary assessment: Convergence of momentary reports of psychotic and mood symptoms with diagnoses and standard clinical assessments. Innovations in Clinical Neuroscience, 18(1–3), 24–30. Retrieved from https://pubmed.ncbi.nlm.nih.gov/34150360/. PMC8195558 34150360

[ref24] Heerey, E. A., & Gold, J. M. (2007). Patients with schizophrenia demonstrate dissociation between affective experience and motivated behavior. Journal of Abnormal Psychology, 116(2), 268–278. doi: 10.1037/0021-843x.116.2.268 17516760

[ref25] Ito, T. A., & Cacioppo, J. T. (2005). Variations on a human universal: Individual differences in positivity offset and negativity bias. Cognition and Emotion, 19(1), 1–26. doi: 10.1080/02699930441000120

[ref26] Kirkpatrick, B., Strauss, G. P., Nguyen, L., Fischer, B. A., Daniel, D. G., Cienfuegos, A., & Marder, S. R. (2011). The brief negative symptom scale: Psychometric properties. Schizophrenia Bulletin, 37(2), 300–305. doi: 10.1093/schbul/sbq059 20558531 PMC3044634

[ref27] Kraepelin, E. (1921). Dementia praecox and paraphrenia. The Journal of Nervous and Mental Disease, 54(4), 384.

[ref28] Kring, A. M., & Barch, D. M. (2014). The motivation and pleasure dimension of negative symptoms: Neural substrates and behavioral outputs. European Neuropsychopharmacology, 24(5), 725–736. doi: 10.1016/j.euroneuro.2013.06.007 24461724 PMC4020953

[ref29] Kring, A. M., & Moran, E. K. (2008). Emotional response deficits in schizophrenia: Insights from affective science. Schizophrenia Bulletin, 34(5), 819–834. doi: 10.1093/schbul/sbn071 18579556 PMC2632476

[ref30] Lang, P. J., Bradley, M. M., & Cuthbert, B. N. (1997). International affective picture system (IAPS): Technical manual and affective ratings. 1(39–58), 3. doi: 10.1007/978-3-319-28099-8_42-1

[ref31] Larsen, J. T., McGraw, A. P., & Cacioppo, J. T. (2001). Can people feel happy and sad at the same time? Journal of Personality and Social Psychology, 81(4), 684–696. Retrieved from https://pubmed.ncbi.nlm.nih.gov/11642354/. 11642354

[ref32] Larsen, J. T., Norris, C. J., McGraw, A. P., Hawkley, L. C., & Cacioppo, J. T. (2009). The evaluative space grid: A single-item measure of positivity and negativity. Cognition and Emotion, 23(3), 453–480. doi: 10.1080/02699930801994054

[ref33] Lee, E., Cha, Y.-J., Oh, J.-H., Hwang, N.-R., Choi, K.-H., & Seo, H.-J. (2018). F58. Community-based multi-site randomized controlled trial of behavioral activation for negative symptoms of individuals with chronic schizophrenia. Schizophrenia Bulletin, 44(suppl_1), S242–S242. doi: 10.1093/schbul/sby017.589

[ref34] Llerena, K., Strauss, G. P., & Cohen, A. S. (2012). Looking at the other side of the coin: A meta-analysis of self-reported emotional arousal in people with schizophrenia. Schizophrenia Research, 142(1), 65–70. doi: 10.1016/j.schres.2012.09.005 23040736 PMC3502689

[ref35] Mairs, H., Lovell, K., Campbell, M., & Keeley, P. (2011). Development and pilot investigation of behavioral activation for negative symptoms. Behavior Modification, 35(5), 486–506. doi: 10.1177/0145445511411706 21746764

[ref36] Miller, M. L., Raugh, I. M., Strauss, G. P., & Harvey, P. D. (2022). Remote digital phenotyping in serious mental illness: Focus on negative symptoms, mood symptoms, and self-awareness. Biomarkers in Neuropsychiatry, 6, 100047. doi: 10.1016/j.bionps.2022.100047

[ref37] Narkhede, S. M., Luther, L., Raugh, I. M., Knippenberg, A. R., Esfahlani, F. Z., Sayama, H., … Strauss, G. P. (2021). Machine learning identifies digital phenotyping measures most relevant to negative symptoms in psychotic disorders: Implications for clinical trials. Schizophrenia Bulletin, 48(2), 425–436. doi: 10.1093/schbul/sbab134 PMC8886590 34915570

[ref38] Norris, C. J., Gollan, J., Berntson, G. G., & Cacioppo, J. T. (2010). The current status of research on the structure of evaluative space. Biological Psychology, 84(3), 422–436. doi: 10.1016/j.biopsycho.2010.03.011 20346389 PMC2894997

[ref39] Norris, C. J., Larsen, J. T., Crawford, L. E., & Cacioppo, J. T. (2011). Better (or worse) for some than others: Individual differences in the positivity offset and negativity bias. Journal of Research in Personality, 45(1), 100–111. doi: 10.1016/j.jrp.2010.12.001

[ref40] Nuechterlein, K. H., Green, M. F., Kern, R. S., Baade, L. E., Barch, D. M., Cohen, J. D., … Marder, S. R. (2008). The MATRICS consensus cognitive battery, part 1: Test selection, reliability, and validity. American Journal of Psychiatry, 165(2), 203–213. doi: 10.1176/appi.ajp.2007.07010042 18172019

[ref41] Onnela, J. P., & Rauch, S. L. (2016). Harnessing smartphone-based digital phenotyping to enhance behavioral and mental health. Neuropsychopharmacology, 41(7), 1691–1696. doi: 10.1038/npp.2016.7 26818126 PMC4869063

[ref42] Perivoliotis, D., Grant, P. M., & Beck, A. T. (2010). Negative symptom focused cognitive behavior therapy: A comprehensive treatment manual. Philadelphia, PA: The University of Pennsylvania.

[ref43] Raugh, I., Howie, S., Gonzalez, C., Chapman, H., Cohen, A., Kirkpatrick, B., … Strauss, G. (2020). Geolocation as a digital phenotyping measure of negative symptoms and functional outcome. Schizophrenia Bulletin, 46, 1596–1607. doi: 10.1093/schbul/sbaa121 PMC7751192 32851401

[ref44] Raugh, I. M., James, S. H., Gonzalez, C. M., Chapman, H. C., Cohen, A. S., Kirkpatrick, B., & Strauss, G. P. (2021). Digital phenotyping adherence, feasibility, and tolerability in outpatients with schizophrenia. Journal of Psychiatric Research, 138, 436–443. doi: 10.1016/j.jpsychires.2021.04.022 33964681 PMC8192468

[ref45] Riehle, M., Pillny, M., & Lincoln, T. M. (2022). Expanding the positivity offset theory of anhedonia to the psychosis continuum. npj Schizophrenia, 8(1), 47. doi: 10.1038/s41537-022-00251-x PMC9261090 35853895

[ref46] Strassnig, M., Miller, M., Moore, R., Depp, C., Pinkham, A., & Harvey, P. (2021). Assessing avolition with ecological momentary assessment. Biological Psychiatry, 89, S140–S141. doi: 10.1016/j.biopsych.2021.02.362 PMC8141033 33848963

[ref47] Strauss, G. P., Raugh, I. M., Zhang, L., Luther, L., Chapman, H. C., Allen, D. N., … Cohen, A. S. (2022). Validation of accelerometry as a digital phenotyping measure of negative symptoms in schizophrenia. npj Schizophrenia, 8(1), 37. doi: 10.1038/s41537-022-00241-z PMC9261099 35853890

[ref48] Strauss, G. P., Visser, K. H., Lee, B. G., & Gold, J. M. (2017). The positivity offset theory of anhedonia in schizophrenia. Clinical Psychological Science, 5(2), 226–238. doi: 10.1177/2167702616674989 28497008 PMC5421554

[ref49] Strauss, G. P., Waltz, J. A., & Gold, J. M. (2013). A review of reward processing and motivational impairment in schizophrenia. Schizophrenia Bulletin, 40(Suppl_2), S107–S116. doi: 10.1093/schbul/sbt197 24375459 PMC3934394

