# Supplementary material for: The positivity offset theory of anhedonia in schizophrenia: evidence for a deficit in daily life using digital phenotyping
Source: Psychol Med. 2023 Feb 1;53(14):6491–9. doi: 10.1017/S0033291722003774 (PMC10600929; doi:10.1017/S0033291722003774)
Supplement: Supplementary file 1 [file S0033291722003774sup001.docx]

THE POSITIVITY OFFSET THEORY OF ANHEDONIA IN SCHIZOPHRENIA: EVIDENCE FOR A DEFICIT IN DAILY LIFE USING DIGITAL PHENOTYPING

Lisa A. Bartolomeo, M.S

Ian M. Raugh, M.S.

Gregory P. Strauss, Ph.D.*

University of Georgia, Department of Psychology, Athens, GA, USA

*Correspondence concerning this article should be addressed to Gregory P. Strauss, Ph.D., Email: gstrauss@uga.edu. Phone: +1-706-542-0307. Fax: +1-706-542-3275. University of Georgia, Department of Psychology, 125 Baldwin St., Athens, GA 30602.

**Supplemental Material**

**Supplemental Data Analysis**

Standard preliminary analyses of self-reported emotional experience variables were conducted similar to past studies comparing subjective positivity, negativity, and arousal to pleasant, unpleasant, and neutral stimuli. The average level of positive affect, negative affect, and arousal were calculated for each participant using responses to surveys completed during positive, negative and neutral contexts and used as the dependent variables for separate Group (SZ, CN) x Emotion Context (Positive, Negative, Neutral) mixed models ANOVAs. Multi-level models were also conducted in R to examine the effects of Group, Emotion Context, and Day on positive affect, negative affect, and arousal. Separate one-way ANOVAs were also used to compare the frequency of positive, negative, and neutral contexts endorsed by SZ and CN participants during the digital phenotyping period.

Exploratory analyses consisted of conducting univariate ANOVA to examine the effects of two between-subjects factors, Sex (Male, Female) and Group (SZ, CN), and the Sex X Group interaction on the positivity offset and negativity bias difference scores, as well as the raw positivity and negativity parameters. Significant interactions were decomposed using one-way ANOVAs. One-way ANOVA was used to examine group (SZ, SZaff, CN) differences in the positivity offset and negativity bias difference scores and raw scores between CN and participants with schizophrenia and schizoaffective disorder. LSD tests were used for post-hoc comparisons between groups. To examine medication effects in the SZ group, exploratory point-biserial correlations were conducted to examine the association between medication status (i.e., whether a participant was prescribed antipsychotics, coded as No = 0 and Yes = 1) and positivity offset and negativity bias difference scores. Lastly, bivariate correlations were used to examine the association between positivity offset and negativity bias scores with cognitive performance measured via the MATRICS Consensus Cognitive Battery (MCCB), positive symptoms measured via the Positive and Negative Syndrome Scale (PANSS), and depressive symptoms measured via the PANSS Depression item.

**Supplemental Results**

***Preliminary Analyses***

Preliminary analyses are displayed in Supplemental Table 1 and Figure 1. Mixed-models ANOVA indicated that for positive affect, there was a significant main effect of Emotion Context (*F*(2, 48) = 40.63, *p* < .001, $\eta_{p}^{2}$ = 0.63), while the main effect of Group (*F*(1, 24) = 1.83, *p* = .19, $\eta_{p}^{2}$ = 0.07) and the Group X Emotion Context interaction were nonsignificant (*F*(2, 48) = .65, *p* = .49, $\eta_{p}^{2}$ = 0.03). Similarly, multi-level models indicated significant effects of Context (*t* = 4.99, *p* < .001), Day (*t* = -2.93, *p* = .003), and the Group x Day interaction (*t* = 3.36, *p* < .001) on positive affect. The effects of Group (*t* = .79, *p* = .43), the Group X Emotion Context interaction (*t* = 1.57, *p* = .12), and the Group x Day x Context interaction (*t* = -.52, *p* = .60) were nonsignificant.

For negative affect, there were significant main effects of Context (*F*(2, 48) = 45.33, *p* < .001, $\eta_{p}^{2}$ = 0.65) and Group (*F*(1, 24) = 5.69, *p* = .03, $\eta_{p}^{2}$ = 0.19), as well as a nonsignificant Group X Context interaction (*F*(2, 48) = 1.04, *p* = .33, $\eta_{p}^{2}$ = 0.04). Multi-level models mirrored these results, yielding significant effects of Group (*t* = -5.15, *p* < .001) and Context (*t* = -5.49, *p* = < .001) on negative affect. The Group x Day (*t* = -.66, *p* = .51), Group x Context (*t* = -.58, *p* = .57), Day x Context (*t* = .15, *p* = .88), and Group x Day x Context interactions were all nonsignificant.

For arousal ratings, there was a significant main effect of Context (*F*(2, 56) = 7.87, *p* = .001, $\eta_{p}^{2}$ = 0.22), while the main effect of Group (*F*(1, 28) = .02, *p* = .88, $\eta_{p}^{2}$ = 0.001) and the Group X Context interaction were nonsignificant (*F*(2, 56) = 1.30, *p* = .28, $\eta_{p}^{2}$ = 0.04). The results of multilevel models indicated nonsignificant main effects and interactions, including Group (*t* = -1.11, *p* = .27), Day (*t* = -.04, *p* = .97), Context (*t* = -.86, *p* = .39), Group x Day (*t* = -.45, *p* = .65), Group x Context (*t* = 1.95, *p* = .05), Day x Context (*t* = 1.21, *p* = .23), and Group x Day x Context (*t* = -.94, *p* = .35).

SZ endorsed significantly fewer neutral contexts throughout the 6-day digital phenotyping period than CN (M_CN_ = 30.25, SD_CN_ = 9.26; M_SZ_ = 23.61, SD_sz_ = 10.86; *F*(1, 58) = 10.00, *p* = .002, $\eta_{p}^{2}$ = 0.10) and significantly more mixed (i.e., positive and negative) contexts (M_CN_ = 1.13, SD_CN_ = 1.62; M_SZ_ = 2.23, SD_sz_ = 2.78; *F*(1, 91) = 5.52, *p* = .02, $\eta_{p}^{2}$ = 0.06). Group differences in the frequency of positive (M_CN_ = 2.52, SD_CN_ = 2.24; M_SZ_ = 3.42, SD_sz_ = 5.75; *F*(1, 44) = .52, *p* = .48, $\eta_{p}^{2}$ = 0.01) and negative contexts (M_CN_ = 2.30, SD_CN_ = 1.75; M_SZ_ = 2.34, SD_sz_ = 1.57; *F*(1, 59) = .01, *p* = .92, $\eta_{p}^{2}$ = 0) were nonsignificant.

***Exploratory Analyses***

The results of univariate ANOVA indicated significant main effects of Group (*F*(1, 92) = 5.31, *p* = .02, $\eta_{p}^{2}$ = .06) and Sex (*F*(1, 92) = .004, *p* = .95, $\eta_{p}^{2}$ = 0) on the positivity offset difference score. The Group X Sex interaction was significant (*F*(1, 92) = 4.74, *p* = .03, $\eta_{p}^{2}$ = 0.05), such that, on average, the positivity offset was larger in females (M = 46.65, SD = 29.53) than males (M = 28.42, SD = 28.42) in the CN group and larger in males (M = 27.31, SD = 46.62) than females (M = 7.92, SD = 46.03) in the SZ group; however, post-hoc one-way ANOVA comparisons indicated that sex differences in the positivity offset were nonsignificant in both CN (*F*(1, 47) = 3.69, *p* = .06, $\eta_{p}^{2}$ = 0.07) and SZ (*F*(1, 43) = 1.79, *p* = .19, $\eta_{p}^{2}$ = 0.04). For the negativity bias difference score, the main effect of Group was significant (*F*(1, 92) = 4.45, *p* = .04, $\eta_{p}^{2}$ = .05) and the main effect of Sex was nonsignificant (*F*(1, 92) = .57, *p* = .45, $\eta_{p}^{2}$ = .01), as was the Group x Sex interaction (*F*(1, 92) = 1.72, *p* = .19, $\eta_{p}^{2}$ = .02). When using the raw positivity slope to represent hedonic capacity, the main effects of Group (*F*(1, 92) = 2.75, *p* = .10, $\eta_{p}^{2}$ = .03), Sex (*F*(1, 92) = .69, *p* = .41, $\eta_{p}^{2}$ = .01), and the Group x Sex interaction were nonsignificant (*F*(1, 92) = 1.99, *p* = .16, $\eta_{p}^{2}$ = .02). When using the raw negativity slope score to represent the negativity bias, the main effects of Group (*F*(1, 92) = 1.91, *p* = .17, $\eta_{p}^{2}$ = .02), Sex (*F*(1, 92) = .09, *p* = .76, $\eta_{p}^{2}$ = .001), and the Group x Sex interaction were nonsignificant (*F*(1, 92) = .32, *p* = .58, $\eta_{p}^{2}$ = .004). See Supplemental Table 2 summary of Group and Sex effects for all positivity and negativity parameters.

Group (SZ, SZaff, and CN) comparisons indicated significant differences in the positivity offset difference score (*F*(2, 92) = 5.54, *p* = .01, $\eta_{p}^{2}$ = .11), such that individuals with both SZ (M = 11.01, SD = 40.65) and SZaff (M = 17.71, SD = 51.01) demonstrated a reduced positivity offset relative to CN (M = 41.72, SD = 30.07). Both SZ (M = 35.11, SD = 32.73) and SZaff (M = 27.16, SD = 28.61) groups also exhibited greater raw negativity intercepts than CN (M = 11.19, SD = 12.60) (*F*(2, 92) = 8.82, *p* < .001, $\eta_{p}^{2}$ = .17). Group differences for all other positivity and negativity parameters were nonsignificant (see Supplemental Table 3).

Correlations between antipsychotic medication status and the positivity offset (*r* = .10, *p* = .55) and negativity bias differences scores (*r* = .29, *p* = .05) were nonsignificant in SZ. Correlations between cognitive performance measured via the MCCB and the positivity offset (*r = -*.12, *p =* .44) and negativity bias difference scores (*r = -*.18, *p =* .24) were nonsignificant. Greater positive symptoms were associated with an elevated negativity bias (*r* = .32, *p* .04) but not the positivity offset (*r* = .03, *p* .86). More severe depressive symptoms were associated with greater reductions in the positivity offset (*r* = -.30, *p* = .05), as well as lower raw positivity intercepts (*r* = -.33, *p* = .03) and slopes (*r* = -.37, *p* = .02). Depressive symptoms were not significantly correlated with the negativity bias (*r* = .23, *p* = .14), or the raw negativity parameters (intercept: *r* = .19, *p* = .22; slope: *r* = .01, *p* = .97).

**Supplemental Table 1**

*Group Comparisons of Momentary Affect and Arousal*

|  | SZ | CN | Test Statistic |
| --- | --- | --- | --- |
| Across Contexts | | | |
| Positive Affect | 49.29 (13.98) | 57.17 (14.74) | *F*(1, 25) = 1.83  *p* = .19, $\eta_{p}^{2}$ = .07 |
| Negative Affect | 34.59 (15.91) | 21.52 (12.00) | *F*(1, 25) = 5.69  *p* = .03, $\eta_{p}^{2}$ = .19 |
| Arousal | 56.30 (19.73) | 55.39 (11.73) | *F*(1, 29) = .02  *p =* .88, $\eta_{p}^{2}$ = .001 |
| Positive Contexts | | | |
| Positive Affect | 67.48 (23.12) | 704.46 (14.23) | *F*(1, 43) = .14  *p* = .71, $\eta_{p}^{2}$ = .003 |
| Negative Affect | 19.42 (22.72) | 9.54 (7.89) | *F*(1, 43) = 4.47  *p* = .04, $\eta_{p}^{2}$ = .10 |
| Arousal | 63.63 (31.61) | 72.50 (20.36) | *F*(1, 43) = .12  *p* = .73, $\eta_{p}^{2}$ = .003 |
| Negative Contexts | | | |
| Positive Affect | 26.78 (22.52) | 39.83 (22.37) | *F*(1, 58) = 2.52  *p* = .12, $\eta_{p}^{2}$ = .04 |
| Negative Affect | 56.94 (19.69) | 37.86 (20.45) | *F*(1, 58) = 8.54  *p* = .01, $\eta_{p}^{2}$ = .13 |
| Arousal | 53.01 (28.77) | 49.13 (20.75) | *F*(1, 58) = .65  *p* = .42, $\eta_{p}^{2}$ = .01 |
| Neutral Contexts | | | |
| Positive Affect | 53.61 (12.86) | 57.23 (16.45) | *F*(1, 81) = 3.48  *p* = .07, $\eta_{p}^{2}$ = .04 |
| Negative Affect | 27.41 (19.75) | 17.15 (12.06) | *F*(1,76) = 20.60  *p* < .001, $\eta_{p}^{2}$ = .22 |
| Arousal | 52.25 (17.56) | 44.55 (18.24) | *F*(1, 91) = 2.80  *p* = .10, $\eta_{p}^{2}$ = .03 |

*Note.* SZ = schizophrenia group; CN = control group.

**Supplemental Table 2**

*Mixed Models ANOVA Results Examining the Effects of Context and Group on Momentary Affect and Arousal*

|  | Within subjects  (Context) | Between Subjects (Group) | Interaction  (Context x Group) |
| --- | --- | --- | --- |
| Positive Affect | *F*(2, 48) = 40.63  *p* < .001, $\eta_{p}^{2}$ = 0.63 | *F*(1, 24) = 1.83  *p* = .19, $\eta_{p}^{2}$ = 0.07 | *F*(2, 48) = .65  *p* = .49, $\eta_{p}^{2}$ = 0.03 |
| Negative Affect | *F*(2, 48) = 45.33  *p* < .001, $\eta_{p}^{2}$ = 0.65 | *F*(1, 24) = 5.69  *p* = .03, $\eta_{p}^{2}$ = 0.19 | *F*(2, 48) = 1.04  *p* = .33, $\eta_{p}^{2}$ = 0.04 |
| Arousal | *F*(2, 56) = 7.87  *p* = .001, $\eta_{p}^{2}$ = 0.22 | *F*(1, 28) = .02  *p* = .88, $\eta_{p}^{2}$ = 0.001 | *F*(2, 56) = 1.30  *p* = .28, $\eta_{p}^{2}$ = 0.04 |

*Note.* Context = positive, negative, or neutral momentary emotional context. Group = SZ or CN.

**Supplemental Figure 1**

*Positivity, Negativity, and Arousal Ratings by Group and Emotion Context*

.

**Supplemental Table 3**

*Univariate ANOVA Results Examining the Effects of Sex and Group on Positivity and Negativity Parameters*

|  | Between subjects  (Sex) | Between Subjects (Group) | Interaction  (Sex x Group) | Post-hoc |
| --- | --- | --- | --- | --- |
| Positivity Intercept | *F*(1, 92) = .02  *p* = .90, $\eta_{p}^{2}$ = 0 | *F*(1, 92) = .55  *p* = .46, $\eta_{p}^{2}$ = .01 | *F*(1, 92) = 3.77  *p* = .06, $\eta_{p}^{2}$ = .04 | - |
| Negativity Intercept | *F*(1, 92) = .06  *p* = .81, $\eta_{p}^{2}$ = .001 | *F*(1, 92) = 9.90  *p* = .002, $\eta_{p}^{2}$ = .10 | *F*(1, 92) = 2.99  *p* = .09, $\eta_{p}^{2}$ = .03 | - |
| Positivity Slope | *F*(1, 92) = .69  *p* = .41, $\eta_{p}^{2}$ = .01 | *F*(1, 92) = 2.75  *p* = .10, $\eta_{p}^{2}$ = .03 | *F*(1, 92) = 1.99  *p* = .16, $\eta_{p}^{2}$ = .02 | - |
| Negativity Slope | *F*(1, 92) = .09  *p* = .76, $\eta_{p}^{2}$ = .001 | *F*(1, 92) = 1.91  *p* = .17, $\eta_{p}^{2}$ = .02 | *F*(1, 92) = .32  *p* = .58, $\eta_{p}^{2}$ = .004 | - |
| Positivity Offset | *F*(1, 92) = .004  *p* = .95, $\eta_{p}^{2}$ = 0 | *F*(1, 92) = 5.31  *p* = .02, $\eta_{p}^{2}$ = .06 | *F*(1, 92) = 4.74  *p* = .03, $\eta_{p}^{2}$ = 0.05 | - |
| Negativity Bias | (*F*(1, 92) = .57  *p* = .45, $\eta_{p}^{2}$ = .01 | *F*(1, 24) = 5.69  *p* = .03, $\eta_{p}^{2}$ = 0.19 | (*F*(1, 92) = 1.72  *p* = .19, $\eta_{p}^{2}$ = .02) |  |

*Note.* Sex = male (M) or female (F). Group = SZ or CN.

**Supplemental Table 4**

*One-way ANOVAs Comparing Positivity and Negativity Parameters in Clinical and Control Groups*

|  | SZ (n=18) | SZaff (n=26) | CN (n=48) | Test Statistic | Post-hoc |
| --- | --- | --- | --- | --- | --- |
| Positivity Intercept | 46.12 (20.25) | 44.87 (28.00) | 52.91 (21.06) | *F*(2. 92) = 1.24  *p* = .30, $\eta_{p}^{2}$ = .03 | - |
| Negativity Intercept | 35.11 (32.73) | 27.16 (28.61) | 11.19 (12.60) | *F*(2, 92) = 8.82  *p* < .001, $\eta_{p}^{2}$ = .17 | SZaff, SZ > CN |
| Positivity Slope | .11  (.49) | -.33  (1.30) | .06  (.26) | *F*(2, 92) = 2.80  *p* = .07, $\eta_{p}^{2}$ = .06 | - |
| Negativity Slope | .45  (1.73) | .18  (1.10) | .03  (.19) | *F*(2, 92) = 1.26  *p* = .29, $\eta_{p}^{2}$ = .03 | - |
| Positivity Offset | 11.01 (40.65) | 17.71 (51.01) | 41.72 (30.07) | *F*(2, 92) = 5.54  *p* = .01, $\eta_{p}^{2}$ = .11 | SZaff, SZ < CN |
| Negativity  Bias | .33  (1.84) | .52  (1.69) | -.03  (.40) | *F*(2, 92) = 1.79  *p* = .17, $\eta_{p}^{2}$ = .04 | - |

*Note.* SZ = Schizophrenia group; SZaff = Schizoaffective group; CN = Control group. Positivity Offset = Positivity Intercept – Negativity Intercept. Negativity Bias = Negativity Slope – Positivity Slope. Values reflect Mean (SD) unless otherwise indicated.
